# The discrete roles of individual FOXO transcription factor family members in B-cell malignancies

**DOI:** 10.3389/fimmu.2023.1179101

**Published:** 2023-05-18

**Authors:** Jamie Lees, Jodie Hay, Michael W. Moles, Alison M. Michie

**Affiliations:** Paul O’Gorman Leukaemia Research Centre, School of Cancer Sciences, University of Glasgow, Glasgow, United Kingdom

**Keywords:** FOXO transcription factor, B cell malignancy, tumor suppressor, BCR signalling, PI3K/AKT, leukemia, lymphoma

## Abstract

Forkhead box (FOX) class O (FOXO) proteins are a dynamic family of transcription factors composed of four family members: FOXO1, FOXO3, FOXO4 and FOXO6. As context-dependent transcriptional activators and repressors, the FOXO family regulates diverse cellular processes including cell cycle arrest, apoptosis, metabolism, longevity and cell fate determination. A central pathway responsible for negative regulation of FOXO activity is the phosphatidylinositol-3-kinase (PI3K)-AKT signalling pathway, enabling cell survival and proliferation. FOXO family members can be further regulated by distinct kinases, both positively (e.g., JNK, AMPK) and negatively (e.g., ERK-MAPK, CDK2), with additional post-translational modifications further impacting on FOXO activity. Evidence has suggested that FOXOs behave as ‘*bona fide*’ tumour suppressors, through transcriptional programmes regulating several cellular behaviours including cell cycle arrest and apoptosis. However, an alternative paradigm has emerged which indicates that FOXOs operate as mediators of cellular homeostasis and/or resistance in both ‘normal’ and pathophysiological scenarios. Distinct FOXO family members fulfil discrete roles during normal B cell maturation and function, and it is now clear that FOXOs are aberrantly expressed and mutated in discrete B-cell malignancies. While active FOXO function is generally associated with disease suppression in chronic lymphocytic leukemia for example, FOXO expression is associated with disease progression in diffuse large B cell lymphoma, an observation also seen in other cancers. The opposing functions of the FOXO family drives the debate about the circumstances in which FOXOs favour or hinder disease progression, and whether targeting FOXO-mediated processes would be effective in the treatment of B-cell malignancies. Here, we discuss the disparate roles of FOXO family members in B lineage cells, the regulatory events that influence FOXO function focusing mainly on post-translational modifications, and consider the potential for future development of therapies that target FOXO activity.

## 1 Introduction

**Figure 1:**
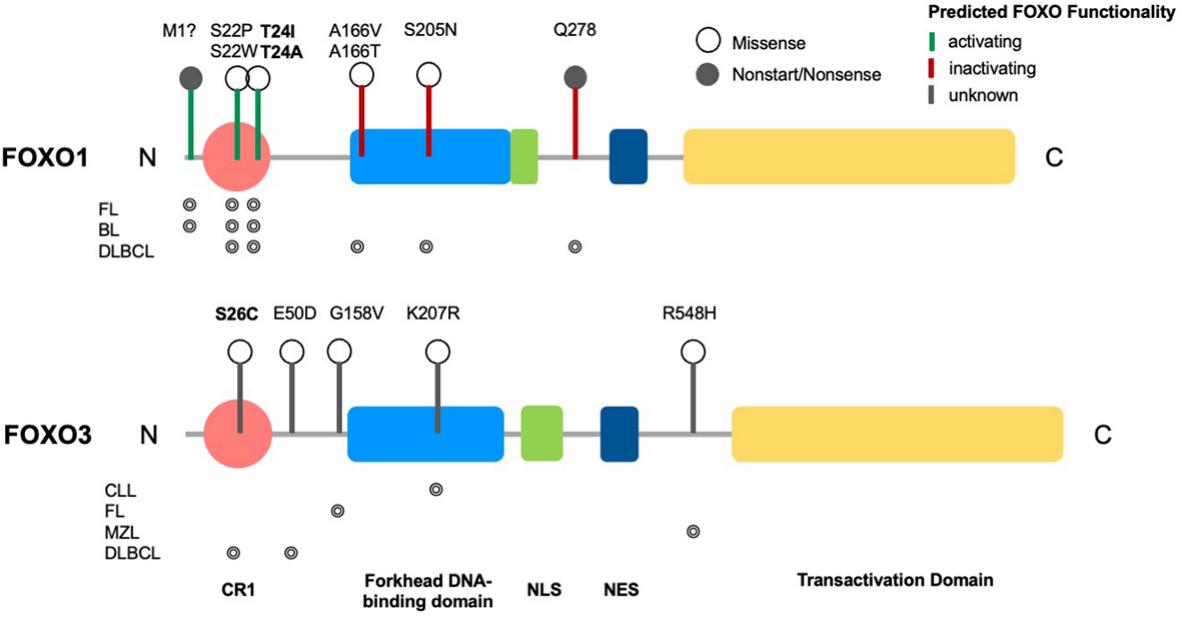
Structure of FOXO transcription factors and common mutations across B cell malignancies. Domain structure of FOXO1 (655 aa) and FOXO3 (673 aa), consisting of a conserved region (CR1), a forkhead DNA-binding domain (DBD), a nuclear localization signal (NLS), a nuclear export sequence (NES) and a C-terminal transactivation domain (TAD). Markers show FOXO1 driver mutations across B cell malignancies (CLL, DLBCL, FL, BL, MZL) and variants of uncertain significance (VUS) in FOXO3, with phosphorylation sites highlighted in bold. Occurrence of respective mutations in FOXO1 and FOXO3 across B cell malignancies are shown below the relevant structural schematic [data from combined studies, 2298 samples (16–18)].

FOXO transcription factors are a family of proteins belonging to a larger superfamily that contains an evolutionarily conserved forkhead domain ordered alphabetically from FOXA to FOXR (the FOX transcription factor superfamily; reviewed in (1)). The FOXO family comprise four highly related members: FOXO1 (FKHR), FOXO3 (FKHRL1), FOXO4 (AFX) and FOXO6 in mammals, which are orthologs of DAF-16 in *Caenorhabditis elegans* and dFOXO in *Drosophila melanogaster.* Mammalian FOXO family members were initially reported as part of pro-oncogenic fusion proteins of paired box protein 3/7 (PAX)-FOXO1 in alveolar rhabdomyosarcoma, whereby FOXO1 trans-activation and the FOXO-dependent TGF-β response was inhibited, thus promoting tumorigenesis (2, 3). Similarly, FOXO3 and FOXO4 form mixed lineage leukemia (MLL) fusion partners, and aggressive paediatric acute leukemia, from translocation of t(6;11)(q21;q23) and t(X;11)(q13.1;q23) respectively (4–6). FOXO family members regulate gene expression through activation or repression. As such, FOXOs are vital for the regulation of a plethora of cellular processes, from cell cycle arrest and apoptosis to metabolism and oxidative damage modulation (7). Structurally, FOXOs differ from the rest of the FOX superfamily, containing a specific amino acid sequence flanking the DNA-binding domain (DBD) (Gly-Asp-Ser-Asn-Ser) enabling interaction with the FHRE (forkhead response element; 5’-GTAAACAA-3’) (8–10). Within the family, FOXOs have shared DBD homology, however the structure of their transactivation domains (TAD) differ; it is this difference in TAD structure that determines the nature of FOXO interactors to define role specificity (11). Partnered with differences in TAD structure are the tissue-specific expression patterns of FOXO isoforms, suggesting FOXOs have cell-specific roles (10). While FOXO1, FOXO3 and FOXO4 family members are ubiquitously expressed, FOXO6 has a more restricted expression pattern. Interestingly, FOXO1-deficient mice are embryonic lethal, dying at day 10.5 of gestation, due to a block in vascular development, while FOXO3- and FOXO4-deficient mice are viable and largely indistinguishable from wildtype mice (12). Therefore, functional redundancy exists between FOXO family members (12–14), with studies suggesting that FOXOs can be overexpressed to fulfil the roles of other family members should they become dysfunctional (15). In addition to TAD sequences, FOXO proteins also contain NLS (nuclear localisation signal), NES (nuclear export signal), all of which are regulated by post-translational modification (**Figure 1** (7, 11)). As FOXOs require nuclear DNA binding to produce cellular effects, FOXO activity is partnered with subcellular localisation.

## 2 FOXO regulation

### 2.1 Akt-mediated regulation of FOXO

In B lymphocytes, B-cell receptor (BCR) ligation is a key signalling component responsible for inducing cell growth and survival (19, 20). Upon crosslinking the BCR with antigen, ITAM motifs on CD79A/B are phosphorylated by Lyn tyrosine kinase enabling the generation of signalling hubs containing adaptor proteins and tyrosine kinases including BLNK, BCAP and Syk. Activated Syk phosphorylates BCAP, which in turn activates PI3Kδ, resulting in conversion of PIP_2_ to PIP_3_ and recruitment of PDK1 and AKT to the plasma membrane. PDK1 phosphorylates and activates Akt^T308^, enabling further phosphorylation/activation of AKT^S473^ by mechanistic target of rapamycin complex 2 (mTORC2 (19, 21)). AKT regulates a wide array of cell functions *via* phosphorylation of target proteins including the FOXO family. Multiple serine/threonine RxRxxS/T regions within the N-terminus, NLS and NES of FOXOs are present, which are conserved from the *C. elegans* protein DAF-16 (FOXO1 - T24, S256, S319; FOXO3 - T32, S253, S315; FOXO4 - T28, S193, S258; FOXO6 - T26, S184. FOXO6 lacks a C-terminal phosphorylation site (7, 15, 22, 23)). AKT-mediated phosphorylation leads to FOXO inactivation by enabling docking of 14-3-3 proteins *via* RSxpS/TxP and RxxxpSxP motifs (24, 25). 14-3-3 proteins can also affect the binding affinity of DNA for FOXOs by interrupting the DNA binding process at the DBD (26). This leads to a conformational change that exposes more of the NES than the NLS, thus preventing FOXO from returning to the nucleus post-translocation (27, 28). Downstream of AKT, mTORC1 also has a pivotal role in inducing positive cellular effects such as protein translation, cell growth and proliferation (29). FOXOs hinder mTORC1 function by inducing *RICTOR* expression, thus reducing RAPTOR-mTOR association, in turn mTORC1 function and preventing cell proliferation (30). FOXOs also downregulate mTORC1 *via SESN3* (sestrin3) upregulation, which in turn activates tuberous sclerosis 1 and 2 (TSC1 & TSC2) *via* AMPK (31). Interestingly, TSC1 and TSC2 can also activate mTORC1 activity if stimulated by other proteins (30), demonstrating the effectiveness of FOXOs in overriding proliferative signals.

### 2.2 Additional mechanisms of FOXO regulation

Although AKT-mediated FOXO phosphorylation is the most understood post-translational modification of FOXO activity, there are other pathways through which FOXO activity is modulated leading to a broad range of biological effects. FOXO localisation (and subsequent transcriptional activity) is negatively regulated by MAPK/ERK, CDK2 and casein kinase 1-mediated phosphorylation (32–36). Conversely, FOXO activity is upregulated by JNK-mediated phosphorylation in response to oxidative stress or AMPK-mediated phosphorylation in response to reduced intracellular ATP, leading to enhanced nuclear localisation and subsequent transcriptional activity, for example through phosphorylation of FOXO1 at Ser^383^ and Thr^649^ (37, 38). In cases such as these, FOXOs respond to intracellular stress stimuli by upregulating genes that maintain homeostasis, such as *GPX1* (oxidative damage) and *PGC1α* (metabolism) (15). FOXOs are also susceptible to post-translational modification *via* acetylation, methylation and ubiquitination (39, 40). Several acetyltranferases, deacetylases, ubiquitin ligases and methyltransferases can either activate or repress FOXO activity through lysine modification, resulting in subsequent changes in DNA binding efficacy, protein interaction effectiveness and overall stability (41–44), while mono- and poly-ubiquitination of FOXO transcription factors impact on protein stability and subcellular localisation (32, 45). miRNAs also regulate the FOXO family post-transcriptionally, such as miR-27a, miR-96 and miR-182 reducing *FOXO1* expression in MCF7 cells by targeting the 3’ untranslated region of *FOXO1* (46).

## 3 The role of the FOXO family in B cell development

### 3.1 Early B cell development

**Figure 2:**
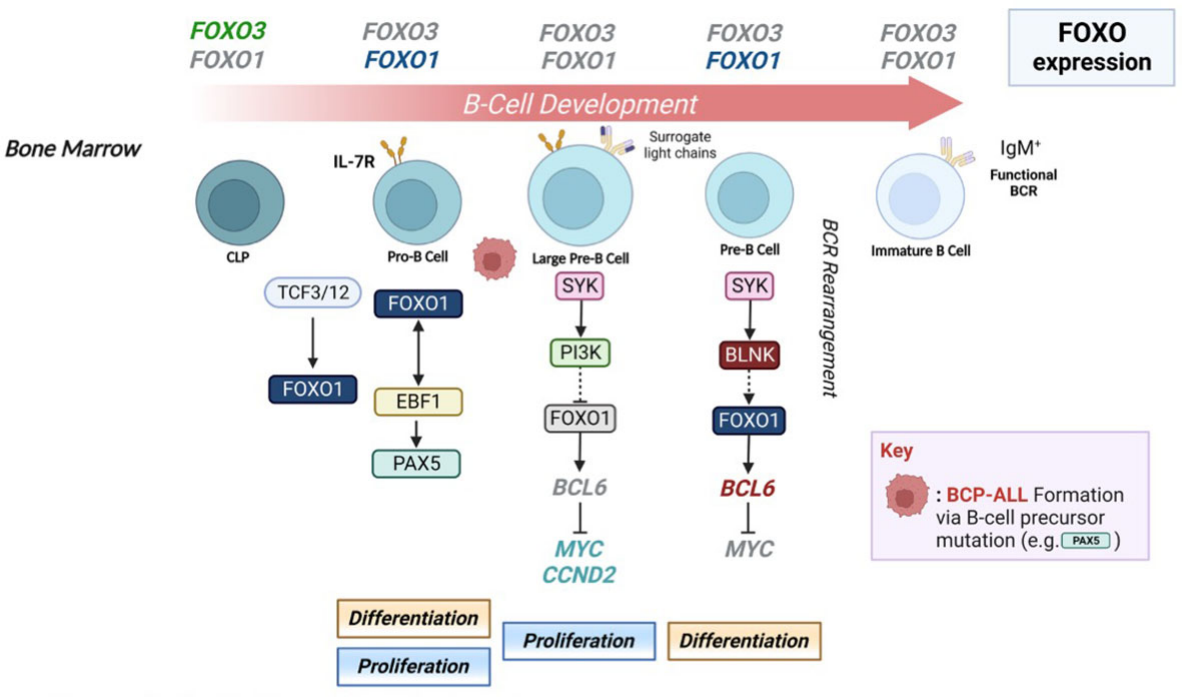
FOXO expression is critical for early B-cell development. B-cells undergo a specific set of developmental stages in the bone marrow (BM), which are tightly controlled by the expression of particular FOXO transcription factors, enabling differentiation and proliferation at distinct stages of lymphopoiesis. *FOXO3* regulates commitment of CLP cells to the B-cell lineage, whereupon *FOXO1* expression enables pro-B cell differentiation via E2A and HEB activity. FOXO1, in conjunction with EBF1, promotes B-cell lineage commitment via activation of PAX5, alongside positive regulators such as IL-7R. Cells advancing to the pre-B cell stage are coordinated by SYK activity, which promotes proliferation or differentiation via activation of PI3K or BLNK respectively. PI3K signalling inactivates FOXO1, leading to an upregulation of *MYC* and *CCND2* expression driving pre-B cell proliferation, while BLNK induces cell cycle arrest via FOXO1 and *BCL6* upregulation. *FOXO1* expression is ablated to allow for differentiation of small pre-B cells into immature B-cells primed to leave the BM to further mature and differentiate in GC reactions. Figure produced in BioRender.

Selective FOXO family members play important roles at distinct stages during B-cell lineage commitment and development (**Figure 2**). During the initial stages of B cell lineage commitment, *FOXO1* enables the differentiation of common lymphoid progenitor (CLP) cells towards the pro-B cell stage. This is initiated by E2A and HeLa E-box binding (HEB) proteins, with the ablation of these proteins diminishing *FOXO1* expression and inducing a block at the CLP stage (47, 48). The transition from the pro-B to pre-B cell stage is dependent on the generation of a functionally rearranged immunoglobulin heavy chain µ (µIgH), surrogate light chains (VPREB and IGLL1) and the signalling components CD79A and CD79B, to form the pre-BCR (49). Pre-B cells further divide and rearrange the Ig light chains (Igκ or Igλ) to generate a functional, mature BCR (50).

FOXO1 specifically plays a critical role during these stages of development through transcriptional activation of the recombination-activating gene (RAG) proteins RAG1 and RAG2, which are responsible for initiating Ig gene rearrangement (50–52). In addition, positive regulators of early lymphopoiesis (IL7R, SYK, PI3KCα and VPREB1/3) are regulated by and can regulate FOXO1 activity (52, 53). During the pre-B cell stage, SYK inactivates or activates FOXO1 *via* downstream activation of PI3K or BLNK respectively (53). An absence of *FOXO1* during this stage prevents somatic recombination in pro-B cells and reduces Ig light chain rearrangement in pre-B cells, while an absence of *FOXO3* has no effect (52). Interestingly, a loss of FOXO1 in pro-B cells also provokes increased levels of apoptosis linked to an increase in the expression of *BCL2L11* (BIM) and lowered *BCL2L1* (BCL-XL) expression (52). These studies highlight a key role for FOXO1 in driving early B cell differentiation and supporting pro-B cell survival.

FOXO1 can evoke further positive or negative impacts on pre-B cell proliferation through upregulation of *CCND3* or *BCL6* respectively. *CCND3* (Cyclin D3) is a FOXO target and crucial for pre-B cell proliferation (54). BCL6 is a repressor of DNA recombination-induced cell death (induced by p53 downregulation) but also prevents proliferation *via* MYC/CCND2 repression (55, 56). This temporal regulation can lead to cell cycle arrest of pre-B cells (57). These observations demonstrate the significant role of FOXO1 in early B-cell development. However *FOXO3* and *FOXO4* are also expressed during development (55). Although the role of *FOXO4* expression in B-cell development is unclear, evidence is emerging for the significance of *FOXO3*, as FOXO3^-/-^ mice exhibit a loss of the pre-B cell population, and a reduction of B cells in the bone marrow (BM) and peripheral blood (58). Although the loss of FOXO1 impaired B cell lineage commitment, studies using a FOXO3 conditional deletion model demonstrated that *FOXO3* deletion affected the production of LMPPs, CLPs and B-cell precursors (59). Furthermore, deletion of both FOXO1 and FOXO3 results in a complete block of CLP commitment towards the B-cell lineage (59). These data shed light on the importance of FOXO3, in addition to FOXO1, in normal B-cell development and indicate a predominant switch of *FOXO3* to *FOXO1* expression is pivotal for B-cell lymphopoiesis (**Figure 2**).

### 3.2 The role of FOXOs in mature B-cells

Mature B-cells express both FOXO1 and FOXO3. Upon BCR crosslinking and downstream PI3K-AKT activation, FOXO1 is downregulated and inactivated (60, 61). There is conflicting evidence regarding the function of FOXO1 in mature B cells. Srinivasan et al., demonstrated that FOXO1 induces apoptosis in response to a lack of BCR signalling, partnered with elevated *BCL2L11* and *CDKN1B* FOXO target expression (62). This was supported by studies in which constitutively active FOXO1 (FOXO1-A3) promoted apoptosis and cell cycle arrest in B-cells (55). However, *FOXO1* expression also supports B-cell populations: conditional deletion of *FOXO1* (*CD21-Cre^+^*) reduced B-cell lymph node populations, presenting FOXO1 expression as being crucial for correct B cell migration (52). *FOXO1* deletion resulted in a reduced capacity for B cells to induce BCR signalling and effectively proliferate (52, 61). The role of FOXO3 in mature B cell maintenance and regulation is also conflicting; constitutively active FOXO3 increased levels of cell cycle arrest and apoptosis in B-cells (61), suggesting a pro-apoptotic function for FOXO3 in mature B-cells. On the other hand, *FOXO3^-/-^* Eμ-myc transgenic mice were shown to have accelerated levels of B-cell lymphomagenesis (63), suggesting that FOXO3 is required for normal B-cell maintenance and regulation. These data demonstrate the context-specific bimodality of FOXO function, with FOXO activity either hindering or promoting cell growth and proliferation.

**Figure 3:**
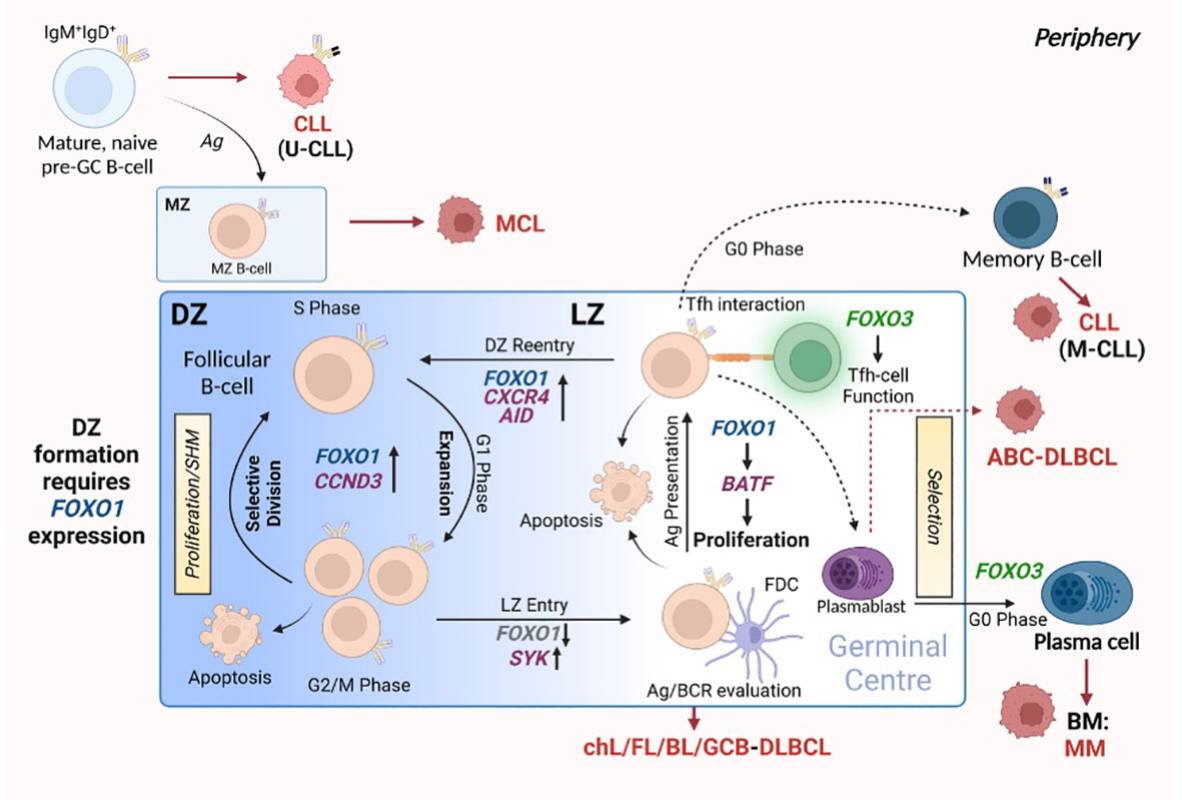
FOXO expression assists in germinal centre (GC) B-cell differentiation. The formation of the GC is crucial for the generation of B-cells that produce high affinity antibodies towards specific antigens and differentiate into plasma cells (PCs) and memory B-cells. This occurs through GC-centralised processes: SHM, affinity maturation and clonal expansion. GCs consist of two distinct compartments: the DZ, in which B-cells cycle between proliferation and SHM, and the LZ, in which cells undergo evaluation and selection processes. *FOXO1* expression is critical for forming and retaining cells in the DZ. Cells then enter the LZ, where the modified/mutated antibody is tested for high-affinity towards antigen: this process requires *FOXO1* downregulation via SYK. Here, B-cells interact with FDCs to evaluate antigen affinity and BCR function, while Tfh-cell interactions aid in B-cell differentiation and proliferation by providing the appropriate signals. Of note, the LZ requires specific regulation of *FOXO1* expression to allow for correct LZ proliferation via *BATF* induction. B-cells from the LZ gain re-entry into the DZ to undergo clonal expansion regulated by *FOXO1* and *CCND3*. All the while, *FOXO3* expression maintains GC Th-cell populations and allows for the differentiation of PCs. Red arrows and malignant-like cells indicate stages in GC B-cell differentiation where B-cell malignancy can originate. Figure produced in BioRender.

Germinal centres (GC) are specialised structures within secondary lymphoid organs (SLO) in which B-cells undergo clonal expansion and somatic hypermutation (SHM), leading to the generation of antibodies that possess a higher affinity for antigen (affinity maturation) and the export of long-lived plasma cells (PC) and memory B cells (**Figure 3** (reviewed in 61)). Upon initial recognition of antigen, B cells (pre-GC B cells) congregate at the border between the follicle and the T cell zone and undergo proliferation, which leads to the development of the GC structure. GCs comprise a proliferation-rich dark zone (DZ), in which B-cells undergo SHM and clonal expansion, and a light zone (LZ), in which B-cells (centrocytes) undergo selection processes: the new antibody is tested for interaction with follicular dendritic cell bound antigen and receive help from follicular helper T (Tfh) cells (64, 65). Following positive selection of B cells expressing higher affinity antibodies, the B-cells re-enter the DZ and proliferate further, driven by cyclin D3 (66, 67). Activation-induced cytidine deaminase (AID) is an essential component of affinity maturation due to its ability to initiate SHM (68, 69). While introducing nucleotide changes in the variable regions of *Igh*, processing of these mutations can induce DNA double stranded breaks which leads to class switch recombination (CSR) resulting in the generation of IgG, IgA and IgE antibodies. However, evidence indicates that CSR mainly occurs earlier in pre-GC cells (70). Bcl-6 also plays a vital role within the GC B cell program, modulating *Myc* and *Prdm1* (encoding Blimp1) expression which, together with FOXO1 activity, assist in normal GC function (71–73). Indeed, FOXO1 is a critical component in the DZ phenotype, as indicated by the finding that deletion of FOXO1 in GC B cells results in a loss of the anatomical structure of the DZ (73, 74). This happens in part because of the absence of CXCR4 assisted B cell migration: FOXO1-mediated *CXCR4* expression ensures the retention of B-cells in the DZ (73). While *FOXO1* knockdown in GC-B cells prevents DZ formation and inhibits CSR, SHM and clonal expansion are unaffected suggesting that FOXO1 may act to delay the LZ transcription program (52, 73, 74). Supporting these findings, an enhancement in CSR was noted in B cells with nuclear sequestered FOXO1^T24A^ *via* subsequent *AICDA* (AID) upregulation, while inactivation of FOXO1 through AKT-mediated signals led to IRF4-driven PC differentiation (75). In addition to FOXO1 function being required for DZ B-cell populations and GC anatomical formation, FOXO1-mediated induction of *BATF* is required for effective LZ B-cell proliferation (76), demonstrating a need for *FOXO1* expression in both GC compartments. Of note, FOXO1 plays a key role in positive GC B-cell selection, as BCR signalling is programmed to signal *via* FOXO1 activity, while CD40 ligation induces NF-κB activity: both CD40- and BCR-mediated signals are required to induce c-Myc upregulation thus promote B-cell survival (77). Further, FOXO1 induces upregulation of *CCND3* in the GC DZ B cells, enabling GC B cell expansion (66). Collectively, these findings demonstrate that FOXO1 plays a central role in regulating GC processes.

FOXO3 is also important in B-cell maturation through Tfh cell function, an essential cellular component of GC formation (65, 78). *FOXO3*-deficient mice exhibit lower levels of IL-21, anti-ovalbumin antibodies when challenged with ovalbumin, and decreased Tfh and B-cell populations. Additionally, there is a marked reduction in the occurrence of T cell co-stimulator (ICOS)-induced Tfh differentiation (78). *FOXO3* is strongly expressed in B cells committed to PC differentiation (65). Thus, FOXO1 and FOXO3 play inverse roles, both being critical in facilitating mature B-cell differentiation as part of the adaptive immune response.

## 4 The differential roles of FOXOs in B-cell malignancies

FOXOs are traditionally regarded as tumour suppressors due to their canonical activity being associated with detrimental cellular fate (e.g. cell cycle arrest and apoptosis (15)). Indeed, early studies demonstrated that the conditional triple *FOXO1/3/4* deletion in adult mice resulted in the development of thymic lymphomas and hemagiomas (14). However, in line with FOXO family members playing an important physiological role in maintaining self-renewal in stem cell compartments (79), more recent studies revealed that FOXO family members can maintain leukaemia-initiating cells in myeloid leukemias (AML and CML), and promote breast tumour invasion, suggesting that in certain cellular contexts FOXOs play a tumour-promoting role (80–82). The paradox of FOXO proteins driving the inhibition or promotion of cancer development in specific contexts, and the complex regulatory mechanisms that subvert FOXO function in malignant cells identifies the need for a deeper understanding of the cellular and molecular function of these proteins in specific cancers.

**Figure 4:**
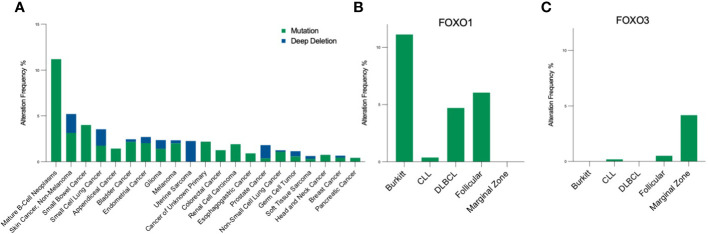
Pan-cancer frequency of FOXO mutations. **(A)** The frequency of *FOXO1* mutations across malignancies [data from MSK-IMPACT, 10945 samples; (83)] highlight that *FOXO1* mutations are most prevalent in B-cell neoplasms. Within the B-cell malignancy subset, **(B)** *FOXO1* is most frequently mutated in Burkitt's lymphoma, and **(C)** *FOXO3* is most frequently mutated in marginal zone lymphoma. Mutations encompass, in-frame, missense and truncating mutations, and deep deletion indicates a deep loss, possibly a homozygous deletion.

FOXO signalling is often compromised and exploited during B-cell development and maturation to promote proliferative and anti-apoptotic signals, providing malignant cells with the means to bypass checkpoints preventing classical cancer hallmarks. Typically, this occurs in a disease-specific context, where FOXOs can exhibit bipartite behaviours, either aiding or preventing tumourigenesis. Of note, somatic point mutations of FOXO1 occur more frequently across B cell malignancies than other cancers, and within B cell malignancies occur predominantly in Burkitt’s lymphoma (BL), follicular lymphoma (FL) and diffuse large B cell lymphoma (DLBCL), with a frequency of ~11%, 6% and ~5% respectively (**Figures 4A, B** (16–18, 83)). Within these diseases, six point mutations have been identified as driver mutations of oncogenesis, where drivers are defined as mutations, fusions and copy number alterations in OncoKB (84) or CancerHotspots (85), with mutations occurring mostly in the N-terminus or forkhead DBD within exon 1 (**Figure 1**). All six driver mutations are considered to be oncogenic through either: gain of function, as demonstrated in *in vitro* studies, which showed escape from PI3K/AKT regulation through loss of AKT phosphorylation; increased nuclear sequestration and increased DNA binding compared to wildtype (86), or; loss-of function, identified through preserved AKT phosphorylation, cytoplasmic retention and decreased transcriptional activity compared to wildtype (87, 88). Additionally, around forty missense and in-frame deletion mutations of *FOXO1* have been identified within B cell malignancies, which are considered variants of uncertain significance (VUS). Mutations of the other FOXO family members are less frequent within B cell malignancies (**Figure 4C**). No driver mutations have been characterised within *FOXO3*, however five missense VUS mutations have been identified within exon 1, with one mutation affecting a putative phosphorylation site, Ser^26^ within marginal zone lymphoma, DLBCL, chronic lymphocytic leukemia (CLL) and BL (**Figure 1**) (89). It should be noted that the 6q21 region encoding *FOXO3* is frequently deleted in DLBCL (with deletions of 6q21-q22 occurring in 40% of ABC-DLBCL cases and 22% of GCB-DLBCL cases) (90) and is associated with adverse prognosis (91). Deletions of 6q21 also occur in mantle cell lymphoma (MCL), FL, acute lymphoblastic leukemia (ALL) and CLL (90, 92).

### 4.1 B-cell precursor acute lymphoblastic leukemia

B-cell precursor acute lymphoblastic leukemia (BCP-ALL) is the most common childhood malignancy originating from pro-B and pre-B cells (49, 93). BCP-ALL arises as a result of a B-cell differentiation block, dysregulation of proliferation and the promotion of survival signals (94). *FOXO1* expression is not altered in BCP-ALL (95, 96). In BCR^+^ BCP-ALL cell lines, pharmacological SYK inhibition blocks FOXO1^T24^ phosphorylation, leading to increased FOXO1 activity and enhanced cell death. Supporting this, constitutively active FOXO1 increases cell death (97). Bhansali et al. demonstrated that the kinase DYRK1A is overexpressed and crucial for BCP-ALL progression; inhibition of DYRK1A and subsequent activation of FOXO1 led to DNA-damage-mediated cell death (98). In contrast, genetic and pharmacological inactivation of FOXO1 produces a tumour suppressive effect by means of caspase-induced cell death in pre-BCR^-^ and pre-BCR^+^ BCP-ALL cells *via CCND3* downregulation (99, 100). *FOXO3* is typically deleted in BCP-ALL (90). In mouse models, FOXM1 is negatively regulated by FOXO3 which promotes cell growth *via* cell cycle progression (101). Interestingly, patient-derived BCP-ALL cells with highly expressed levels of *FOXM1* are associated with poor prognostic outcomes; this data coincides with reduced *FOXO3* expression (102). This suggests BCP-ALL promotes cell growth and survival by means of bypassing the expression of tumour suppressive components such as *FOXO3*.

### 4.2 Chronic lymphocytic leukemia

CLL is characterized by expansion of small, monoclonal CD5^+^ B-lymphocytes in the blood, BM and SLOs (103). FOXO family members play a vital role in CLL biology, with regulation of individual family members impacting on CLL survival. FOXO family mutations occur infrequently in CLL patients, with analysis of the ICGC database (containing data from 551 CLL patients) showing that single base substitutions within *FOXO* family genes occurred in 4% (22/551) of CLL patients, of which 2% (11/551) occurred in *FOXO1*, with a functionally “high impact” missense mutation of *FOXO1* encoded in just one patient (104). Therefore the tumour microenvironment has a more significant impact on the activity of FOXO family members in CLL. FOXO3 is inactivated by chemokine expression (CCL19, CLL21, CXCL12, CXCL13) leading to subsequent BIM downregulation and increased cell survival. Expression of constitutively active FOXO3 increased levels of cell death whereas a reduction in FOXO3 expression increased cell survival, providing evidence for FOXO3 playing a tumour suppressor role in CLL cells (103). Palacios et al. showed that CLL cells required elevated levels of miR-22 expression for effective proliferation, with miR-22 leading to decreased PTEN and increased PI3K-AKT signalling, particularly in poor prognostic patient cohorts. Subsequent FOXO1 inhibition coincided with reduced levels of *CDKN1B* (105). We have demonstrated that while FOXO1 expression was upregulated in CLL cells compared with B cells from healthy donors, it appeared to be inactive, perhaps due to tonic CLL-BCR signalling. However, pharmacological inhibition of PI3K-mTOR/AKT signalling reduced FOXO1 phosphorylation, increased FOXO1 nuclear localisation and resulted in an upregulation of FOXO1-mediated genes, ultimately inducing CLL cell death (106). These studies indicate that FOXO1 is being prevented from performing a tumour suppressor role due to tumour microenvironmental factors supporting the CLL cells, similar to that noted for FOXO3 (103). In contrast, in an aggressive CLL mouse model, FOXO1 was shown to be a driver of disease through induction of *IGF1R* in PI3K-inhibitor-resistant SLO tumours: this was attenuated by pharmacological FOXO1 inhibition (AS1842856) (107). Interestingly, FOXO1 has also been reported to induce *GAB1* which aids in maintenance of CLL cell survival through sustained basal AKT phosphorylation (108). These studies suggests that a low level of FOXO1 activity, possibly promoted through tonic BCR signalling, sustains CLL cell survival, while higher FOXO1 activity induced by inhibition of upstream PI3K/AKT signals through drug treatment triggers CLL cell death (106, 108). Interestingly, a recent study demonstrated that hyperactivation of PI3K/AKT activity mediated by SHIP1 inhibition, can induce CLL cell death (109). While this study did not directly address the involvement of the FOXO family in the induction of cell death, it suggests that the strength of signal upstream of FOXO may regulated the threshold of FOXO activity induced, which in turn impacts on cell fate decisions within CLL. Of note, discrete tumour microenvironments are also likely to play a major role in modulating FOXO activity.

### 4.3 Mantle cell lymphoma

MCL is a rare B-cell malignancy caused by the overexpression of cyclin D1 in B-cells inhabiting the mantle zone surrounding GCs. Studies suggest a tumour suppressive role for FOXO3, as pharmacological inhibition of PI3K-AKT-mTOR signalling resulted in dephosphorylation and activation of FOXO3 leading to a reduction in MCL cell survival and proliferation (110). Additionally, FOXO3 is constitutively inactive and cytoplasm-localised in MCL. Recent evidence however suggests that FOXO1, not FOXO3, acts as a “master regulator” driving MCL progression, with FOXO1 inducing the expression of genes governing MCL lineage survival (111). A novel inhibitor of FOXO1 activity cpd10, blocked MCL progression *in vivo* (111). These data highlight differential roles for FOXO1 and FOXO3 in MCL.

### 4.4 Follicular lymphoma

FL originates in the GC from activated centroblast and centrocyte B-cells defined by genomic aberrations including the t(14,18) translocation resulting in the overexpression of BCL-2 (112). Somatic point mutations suppressing FOXO1 DNA binding activity identified in DLBCL are evident in FL (87). The nonsense mutation at the M1 residue, at the N terminus, leads to a truncation of FOXO1 and loss of the AKT recognition motif, resulting in increased nuclear localisation and transcriptional activity (**Figure 1** (113)). This in turn promotes pro-proliferative and anti-apoptotic signalling and lymphomagenesis (114). However, the FOXO1 mutations drive altered transcriptional profiles which lead to positive selection of GC B-cells (86). Therefore, GC B-cells expressing FOXO1^M1^ expand abnormally with a competitive advantage over normal GC B-cells, and the altered signalling identified in the VavP-*Bcl2* transgenic mouse model of human FL, promotes lymphomagenesis (86). While FL cells exhibit abnormal function of B-cell epigenetic regulators (EZH2, HIST1H1), they also incorporate genetic defects in multiple pathways affecting B-cell growth and development, such as PI3K/AKT and mTOR, suggesting mutation is a key factor through which FL cells bypass screening for correct growth (112). Downstream of PI3K-AKT/mTOR signalling, Pastore et al. demonstrated an association between shorter failure-free survival and *FOXO1* gene mutations, identifying *FOXO1* mutation as a potential biomarker for a model determining FL disease risk (M7-FLIPI) (112, 115). Moreover, venetoclax (BCL2i)-resistant FL cells exhibit increased levels of p-FOXO1/p-FOXO3 associated with diminished BIM activity, thereby preventing cell death supporting the role of FOXO family members as tumour suppressors in FL (116).

### 4.5 Burkitt’s lymphoma

Activity of the *MYC* oncogene is the focal point driving BL pathogenesis (117). As FOXOs are known to negatively regulate *MYC*, FOXOs were considered to have tumour suppressive properties in BL. Supportive work by Bouchard et al. demonstrated an acceleration of BL tumourigenesis following either FOXO3 knockdown or expression of a FOXO3 dominant negative construct in the Eµ-Myc mouse model (118). However, mutations forcing FOXO1 nuclear accumulation can promote BL cell proliferation and survival. Within sporadic BL, mutations of Thr^24^ are most common, while missense mutations of Ser^22^ are most frequent in endemic disease (119, 120). Phosphorylation of Ser^22^ by AMPK directly prohibits the binding of 14-3-3 proteins through steric hindrance and electrostatic repulsion, and indirectly through negative regulation of Thr^24^, therefore mutations of this residue are likely to lead to inactive FOXO1 function and cytoplasmic localisation (120). This mutation is also prevalent in DLBCL and FL (**Figure 1** (16, 17)). CRISPR/Cas9-mediated *FOXO1* ablation strongly restricted tumour growth, demonstrating FOXO1 as a key driver of BL (114). Gehringer et al. demonstrated a loss of BL cell proliferative capacity following *FOXO1* knockdown, supported by an increase in cell death following AS1842856-mediated FOXO1 inhibition (121). Thus, these data provide interesting evidence for FOXO1 behaving as a driver of disease while FOXO3 behaves as a tumour suppressor in BL.

### 4.6 Diffuse large B-cell lymphoma

DLBCL is a heterogeneous disease which was transcriptionally divided into molecular subtypes based on cell of origin: normal GC B-cells (GCB-DLBCL) or activated B-cells (ABC-DLBCL), with ABC-DLBCL associated with an inferior outcome (122). These subtypes have since been sub-divided into five distinct DLBCL subsets, based on the expression of driver mutations, somatic copy number variations and structural variations, which have enabled a more detailed landscape of DLBCL pathogenesis to be appreciated (123). Patients with relapsed/refractory disease (rrDLBCL) have poor disease outcome, with around 40% of patients achieving a longer term remission due to salvage chemotherapy or stem cell transplant (124). The genetic profile of *de novo* DLBCL is well characterised, with common genomic alterations, including chromosomal translocations and mutations found between subtypes, contributing to promotion of pro-survival pathways (125). Within DLBCL, *FOXO1* missense mutations have been reported in ~9% cases (113). However, between the two subtypes, there is an enrichment of mutation frequency of some genes, including *FOXO1* in GCB-DLBCL, which is considered to be a driver mutation (86). Furthermore, in rrDLBCL a significant enrichment of *FOXO1* mutations is seen when compared to untreated DLBCL patients (27% vs. 8.6%) (124). FOXO family members have been reported to elicit conflicting tumour promoting or suppressing activities in DLBCL, which may also reflect the mutational burden that is found in DLBCL patient samples. *FOXO1* mutations however, have been associated with poor prognosis and diminished treatment response; *FOXO1* missense mutations affecting the DBD and the Thr^24^ mutation (**Figure 1**), led to poorer overall survival of patients treated with R-CHOP (rituximab, cyclophosphamide, doxorubicin, vincristine, and prednisone) and is associated with poor prognosis in DLBCL (113). This could in part be due to the negative regulation of CD20 expression by FOXO1, which leads to a reduced response to rituximab. Therefore within non-Hodgkin lymphoma, mutations prohibiting AKT-phosphorylation of FOXO1 may represent an important biomarker of rituximab response (126). Within the DBD, Ser^205^ can be mutated within DLBCL, and *in vitro* studies show that this impairs DNA binding capacity, while retaining some transcriptional activity. It has been hypothesised that mutations within the DBD may suppress DNA dependent tumour suppressor activities but maintain pro-lymphogenic FOXO1 functions (87). A further truncating mutation, Gln^278^ residue on exon 2 of FOXO1, has been identified in DLBCL although it is rare (16). The FOXO1^Q278^ gene product lacks the TAD, rendering it transcriptionally inactive and prevents DNA binding but retains the AKT recognition motif (**Figure 1** (127, 128)), suggesting it will be excluded from the nucleus. Of note, Szydlowski et al. confirmed that FOXO1 drives *BIM* and *CDKN1B* transcription following SYK inhibition (using R406), while FOXO1 ablation prevented R406-induced apoptosis and cell cycle inhibition (129).

FOXO4 has also been associated with poor prognosis in DLBCL, identified as an inducer of resistance to doxorubicin and phenylbutyrate. Additionally, *FOXO4* knockdown reduced colony forming ability of DLBCL cell lines (130). FOXO3, however, has been associated with tumour suppression, as indicated by studies showing that prevention of FOXO3 inactivation due to nuclear export reduces levels of ibrutinib resistance in DLBCL (131). Zheng et al. reported low levels of FOXO3 in DLBCL cells compared to healthy B-cells and high levels of miRNA miR-155. Inhibiting miR-155 induced cell death and reduced proliferation, which coincided with higher expression of FOXO3, suggesting FOXO3 possesses tumour suppressive qualities in DLBCL (132). Collectively, specific FOXOs may differentially contribute to DLBCL disease progression, with FOXO1 and FOXO3 activity assisting in promoting DLBCL cell death and preventing resistance to targeted therapies.

### 4.7 Classical Hodgkin’s lymphoma

cHL is thought to originate as a result of the deactivation of particular signalling pathways in activated GC-localised B-cells, such as NF-κB and JAK/STAT pathways (133). FOXO1 is downregulated in cHL cell lines and cHL patient samples. Additionally, constitutively active FOXO1 induced apoptosis and cell cycle arrest, partnered with increased expression of FOXO targets *CDKN1B* and *BCL2L11*. This study concluded that FOXO1 behaves as a tumour suppressor, and that cHL cells actively reduce FOXO1 levels to aid in disease progression (133). Conversely, *FOXO3* levels are higher in cHL than GC B-cells but lower than in differentiated PCs, suggesting FOXO3 expression contributes directly to the cHL phenotype of being an “abortive PC”. Indeed, overexpression of FOXO3 in cell line models activated cHL-inactive PRDM1α (134), a critical transcription factor that drives the PC differentiation program. These findings indicate that FOXO3 downregulation by cHL cells contributes to the cHL phenotype and overall disease progression, identifying FOXO3 as a tumour suppressor in cHL.

### 4.8 Multiple myeloma

MM is a B-cell malignancy characterized by the accumulation of slow proliferating, apoptosis-resistant PCs residing in the BM (135). FOXO1 and FOXO3 have been found to be highly phosphorylated and inactivated, suggesting tumour suppressive functions for FOXOs in MM. As such, targeted inhibition of GSK3 activity increases FOXO1 and FOXO3 activity leading to cell death *via* upregulation of downstream apoptotic factors including Fas ligand (136). More recently, pharmacological AKT inhibition was demonstrated to induce cell cycle arrest and cell death *via* FOXO activation, thus further cementing the tumour suppressive qualities of FOXO family members in MM (137, 138). De Bruyne et al. demonstrated that IGF1 signalling induces MM cell survival *via* downregulation of the pro-apoptotic factor and FOXO target BIM, at least in part through AKT activation and subsequent inactivation of FOXO3 (139). Supporting this, PI3K signal ablation using a selective inhibitor induces cell cycle arrest and apoptosis coinciding with decreases in FOXO phosphorylation (140). In addition to the PI3K-AKT pathway being a viable target for treating MM, previous MM treatments such as bortezomib, a proteosomal inhibitor, has been shown to induce MM apoptosis coinciding with increased FOXO3 expression and activity (141). More recently the nuclear export receptor inhibitor Selinexor has been approved for clinical use for MM and identifies the contribution of FOXO1 and FOXO3 nuclear sequestration as one of its major functions (142–144). Taken together, these data suggest FOXO1 and FOXO3 harbour tumour suppressive qualities in MM.

## 5 Conclusions

FOXOs have traditionally been regarded as tumour suppressors not only due to their canonical activity being associated with detrimental cellular fate (e.g. cell cycle arrest and apoptosis) (15), but also due to deletion of *FOXO1/3/*4 in adult mice leading to tumour formation (14). Supporting this, a number of studies have demonstrated that FOXO function is diminished in B cell malignancies as discussed above, either by reducing FOXO expression (101, 105, 132), or by FOXO inactivation through distinct cellular environments (103, 106, 139). However, FOXO family members can also promote tumorigenesis, and this can occur in a disease- and cell lineage-dependent manner, as observed in B-cell malignancies (107, 111) and in solid tumours; high expression of *FOXO3* is associated with glioblastoma progression, pancreatic ductal adenocarcinoma, and poor survival rates in breast and colorectal cancers (CRC) (145). Furthermore, FOXO mutations are associated with B-cell disease progression, particularly in DLBCL and BL (113, 114), impacting on the transcriptional activity of FOXO family members and implicating a plethora of avenues by which disease can exploit FOXO expression to promote cell survival and proliferation. In addition to the well documented roles of FOXO1 and FOXO3 in regulating B-cell malignancies, lower expression levels of *FOXO4*, and perhaps *FOXO6*, do not preclude these family members having a role in lymphomagenesis. In DLBCL, *FOXO4* expression is important for maintaining colony formation and drug resistance (130). This study aligns with findings that FOXO3 activity is important for maintenance of leukaemia-initiating cells in myeloid leukemias (80–82), and highlights that individual FOXOs may perform specific functions in a lineage-dependent manner.

In the broader context, studies investigating FOXO expression as a prognostic biomarker have contrasting results. In CRC, low expression of FOXO3 is associated with cancer progression in specimens with normal tissue (146), while high FOXO1 expression is associated with good prognosis in prostate and breast cancers (147, 148). In B-cell malignancy, FOXO1 mutations are used as an indicator of FL progression (115). These particular mutations are also evident in BL and DLBCL and are associated with poorer disease outcomes (87) demonstrating a potential use of FOXO1 as biomarker in specific B-cell malignancies. However, in addition to considering the expression levels of specific FOXO family members or their mutational status, as biomarkers, it may be interesting to consider the utility of FOXO activity. While FOXO family members can be inactivated as a result of microenvironmental factors that impinge on the tumour cell, drugs targeting protein/pathways upstream of FOXO can lead to a reactivation and promotion of its tumour suppressor role (97, 106, 110, 129, 149). The resultant change in FOXO activity can be exploited for the development of novel tumour-specific FOXO-gene signatures. Indeed, repression of FOXO1/3-regulated genes in a MM patients has prognostic significance, being associated with reduced overall survival (138). Therefore gaining a deeper understanding of FOXO family regulation, in addition to providing targeting opportunities for the development of novel therapies, may reveal robust FOXO-related pharmacological/prognostic biomarkers to enhance the clinical management and survival prospects of patients.

## Author contributions

JL, JH and MM wrote the manuscript and JL and JH prepared figures. AM reviewed and edited the manuscript. All authors contributed to the article and approved the submitted version.
